# Awake Laser Ablation for Patients With Tumors in Eloquent Brain Areas: Operative Technique and Case Series

**DOI:** 10.7759/cureus.12186

**Published:** 2020-12-20

**Authors:** Sabastian Hajtovic, Alon Mogilner, John Ard, Jose E Gautreaux, Hannah Britton, Girish Fatterpekar, Matthew G Young, Dimitris G Placantonakis

**Affiliations:** 1 Neurosurgery, City University of New York (CUNY) School of Medicine, New York, USA; 2 Neurological Surgery, New York University (NYU) Grossman School of Medicine, New York, USA; 3 Anesthesiology, New York University (NYU) Grossman School of Medicine, New York, USA; 4 Neurosurgery, NYU Langone Medical Center, New York, USA; 5 Radiology, New York University (NYU) Grossman School of Medicine, New York, USA; 6 Neurosurgery, New York University (NYU) Grossman School of Medicine, New York, USA

**Keywords:** laser ablation, laser interstitial thermal therapy

## Abstract

Background

Magnetic resonance imaging (MRI)-guided laser interstitial thermal therapy (LITT) is a minimally invasive treatment modality that has been gaining traction in neuro-oncology. Laser ablation is a particularly appealing treatment option when eloquent neurologic function at the tumor location precludes conventional surgical excision. Although typically performed under general anesthesia, LITT in awake patients may help monitor and preserve critical neurologic functions.

Objective

To describe intraoperative workflow and clinical outcomes in patients undergoing awake laser ablation of brain tumors.

Methods

We present a cohort of six patients with tumors located in eloquent brain areas that were treated with awake LITT and report three different workflow paradigms involving diagnostic or intraoperative MRI. In all cases, we used NeuroBlate® (Monteris Medical, Plymouth, MN) fiberoptic laser probes for stereotactic laser ablation of tumors. The neurologic status of patients was intermittently assessed every few minutes during the ablation.

Results

The mean preoperative tumor volume that was targeted was 12.09 ± 3.20 cm^3^, and the estimated ablation volume was 12.06 ± 2.75 cm^3^. Performing the procedure in awake patients allowed us close monitoring of neurologic function intraoperatively. There were no surgical complications. The length of stay was one day for all patients except one. Three patients experienced acute or delayed worsening of pre-existing neurologic deficits that responded to corticosteroids.

Conclusion

We propose that awake LITT is a safe approach when tumors in eloquent brain areas are considered for laser ablation.

## Introduction

Magnetic resonance imaging (MRI)-guided laser interstitial thermal therapy (LITT) is a minimally invasive procedure used to treat primary and metastatic brain tumors, as well as radionecrosis [1-8]. In LITT, infrared laser light is converted into thermal energy, which ablates target tissue [1, 2]. Real-time MR thermography displays ablation zones with thermal-damage threshold (TDT) lines [6], allowing the neurosurgeon to optimize therapy of target tissue, while minimizing off-target heating of neighboring structures [9].

The goal of LITT is to ablate as much tumor tissue as possible, without inadvertently injuring perilesional tissue. While the thermodynamic properties of the interface of tumor tissue with the surrounding brain often cause the ablation zone to be confined to the tumor [9], inadvertent spread of heat beyond the tumor boundaries is possible and remains a concern, particular in eloquent brain areas [10, 11]. Prior reports have pointed out the risk for permanent neurologic deficit in such cases. Furthermore, the increase in edema that ensues after LITT may result in transient neurologic decline [10, 11].

The risk of thermal injury to peritumoral brain tissue is reduced by implementing several technical advances. First, live MR thermography allows monitoring of spatiotemporal heat spread during LITT. Second, pre-procedure functional MRI and tractography identify cortical and subcortical structures of interest. Third, stereotactic placement of the laser probe, with either frame-based or frameless approaches, ensures accurate targeting [12-14]. Even despite such measures, however, neurologic deficits can arise.

Ultimately, we believe that neurologic assessment during an awake procedure is the optimal way to preserve function. Previously, Laurent et al. reported a technique based on custom-made masks for stereotactic placement of laser probes and non-invasive immobilization of patients during awake LITT [15]. However, such masks introduce additional cost and increase the duration of preoperative preparation. Here, we show head immobilization in conventional head-fixation devices and without the need for custom-made masks is a feasible approach to awake LITT.

## Materials and methods

We retrospectively reviewed a database of LITT patients at our institution. This review was made possible by an IRB-approved study (11-01733), which does not require patient consent. We identified six patients who underwent awake LITT procedures for ablation of brain tumors between July 2017 and May 2020. Population statistics are presented as mean ± standard error.

**Figure 1:**
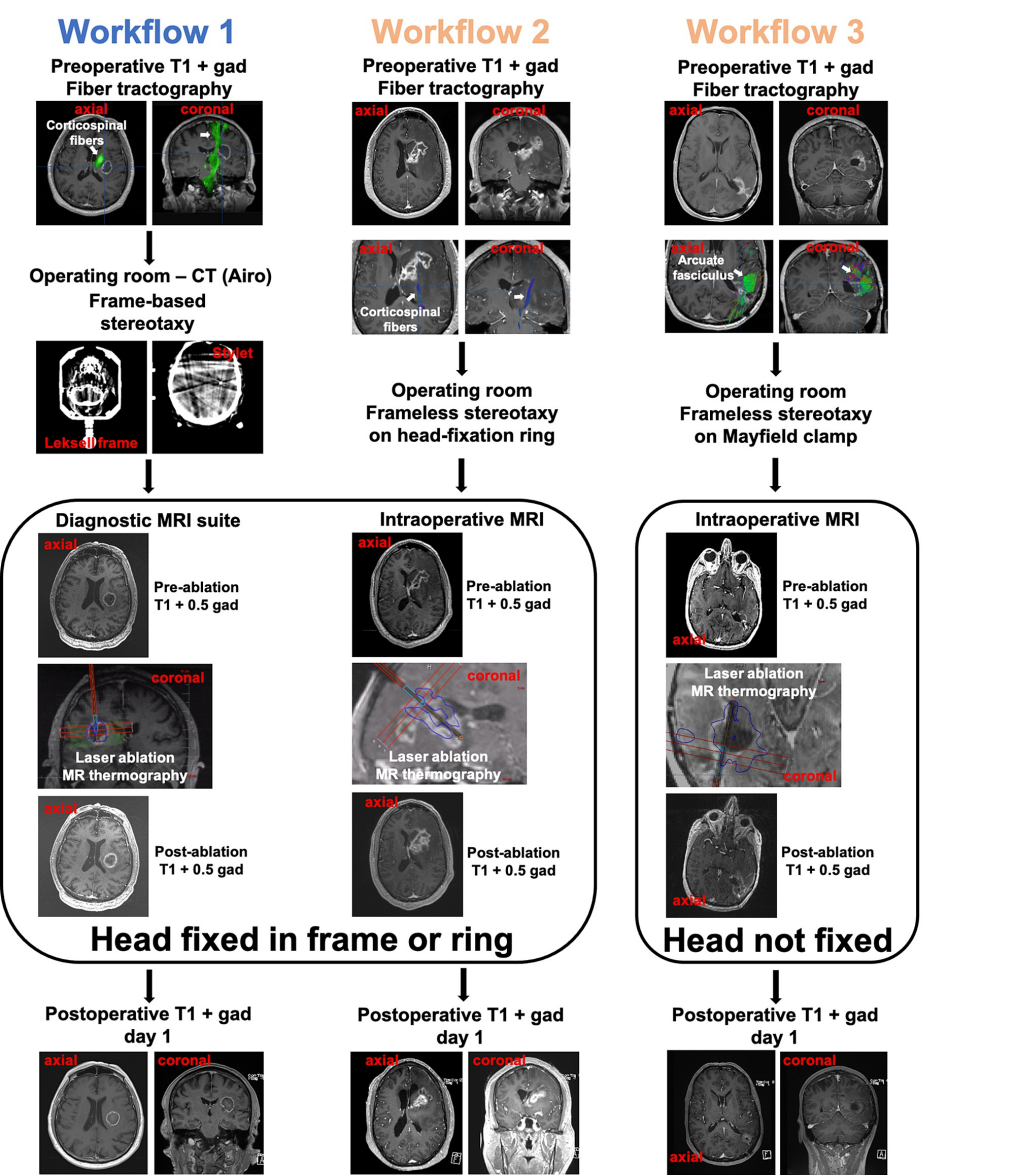
Examples for each of the three workflow paradigms. The example in Workflow 1 shows patient 1, who had a left subinsular isocitrate dehydrogenase (IDH) wild-type glioblastoma (GBM). In workflow 2, patient 5 is shown (left frontal/basal ganglia IDH mutant astrocytoma). In workflow 3, patient 6 is shown (left posterior temporal IDH wild-type GBM). The tractography images highlight the corticospinal tract in patients 1 and 5, and the arcuate fasciculus in patient 6.

We followed three different workflow paradigms (Figure 1), which arose in part from a change at our institution’s facilities and installation of an intraoperative MRI for the last two cases.

In all patients, we used 3.3 mm NeuroBlate® System (Monteris Medical, Plymouth, MN) gas-cooled, fiberoptic laser probes for tumor ablation. In five patients, the diffuse tip probe was utilized, while in one case we opted for the side-firing probe (patient 3).

Anesthesia and sedation

The anesthetic management consisted of light sedation and local anesthesia infiltrated at the sites of head frame or skull clamp pin insertion. At the time of head fixation, all patients received boluses of propofol (0.2-1.0 mg/kg), along with ketamine (0.10-0.25 mg/kg) or fentanyl (0.25-0.50 mg/kg). Pin sites were anesthetized with 0.50% bupivacaine. One patient additionally received a scalp block with 30 ml of 0.25% bupivacaine. The small incision sites were locally anesthetized with lidocaine 1%/epinephrine (1:100,000). Prior to starting the procedure, all patients were administered intravenous antibiotic prophylaxis (cefazolin 2.0 g), prophylactic antiepileptic (levetiracetam 500-1000 mg), and dexamethasone (0-10 mg). Zofran (4.0 mg) was given for postoperative nausea and vomiting prophylaxis.

During the ablation, all patients were mildly sedated with a dexmedetomidine infusion (0.1-0.7 mg/kg/hr). In addition, four patients received a propofol infusion (10-100 mg/kg/min). Finally, remifentanil infusion (0.01-0.06 mg/kg/min, three patients), or ketamine infusion (2-10 mg/kg/min, three patients) was used for comfort.

Workflow 1- Frame-based stereotaxy, intraoperative CT, diagnostic MRI suite, head fixation during ablation

We developed the protocol for patients 1-4 in order to accommodate stereotactic placement of the laser probe in the operating room, followed by laser ablation in a diagnostic MRI suite. Briefly, we placed a stereotactic Leksell Model G frame on the head and attached it to the Trumpf operating table using the Trumpf Radiolucent Bed attachment. We then obtained an intraoperative CT using Airo (Brainlab AG, Germany) imaging. This CT was merged with the preoperative MRI and allowed us to derive stereotactic coordinates for the planned trajectory using BrainLab iPlan Stereotaxy. We generated a stab incision in the scalp and drilled a burr hole with the Stryker πdrive+ in alignment with the planned trajectory. The Monteris Mini Bolt was screwed into this burr hole. After puncturing the dura, we placed a radiopaque stylet with its tip at the tumor target. We then repeated intraoperative CT imaging with Airo, to ensure that the trajectory we had generated aligned with the target. That was indeed the case with all four patients. After removing this stylet, we placed the robotic probe driver (RPD) over the Mini Bolt and passed the fiberoptic NeuroBlate probe through the RPD to the desired depth. The sterile drapes were removed. The bolt and RPD were covered with sterile gauze. The RPD and probe cables were taped to the patient’s chest. Patients were then transferred onto the AtamA® System patient board (Monteris) mounted on an MRI stretcher.

After transport to the diagnostic MRI suite, patients were placed on the MRI (3T MAGNETOM Skyra, Siemens) table, while still on the AtamA® board. The Leksell frame was secured to the frame adaptor on the MRI table. We connected the cables to the laser power source and RPD control station. Following a volumetric T1 MPRAGE scan with half-dose of gadolinium that confirmed correct placement of the NeuroBlate probe within the target, we used near real-time MR thermography and the Monteris proprietary software to monitor the ablation.

After the ablation, an additional T1 MPRAGE sequence with half-dose of gadolinium was obtained. Patients were then transferred to a designated area adjacent to the MRI suite. Under sterile conditions, the probe and bolt were removed, and the stab incision was closed with 3-0 Vicryl Rapide or 3-0 Nylon suture. The incision was covered with sterile dressing and the Leksell frame was removed using standard techniques.

Workflow 2 - Frameless stereotaxy, intraoperative MRI, head fixation during ablation

The procedure for patient 5 was performed in the operating room with intraoperative MRI. The patient was positioned on the AtamA® board, which was mounted on the operating table and allows for frameless stereotaxy. The head was immobilized in the head-fixation ring on the AtamA® board and remained fixed throughout the procedure. We employed frameless stereotaxy to place the Mini Bolt along a preplanned trajectory using the BrainLab VarioGuide mounted on the AtamA® board. No intraoperative CT was necessary. After covering the Mini Bolt with a sterile cap, the sterile drapes were removed and the bolt was covered with sterile gauze. The patient was transported to the intraoperative MRI (3T MAGNETOM Skyra, Siemens), where under sterile conditions we placed the RPD and the NeuroBlate probe through the Mini Bolt. Subsequent T1 MPRAGE sequence with half-dose of gadolinium confirmed accurate placement of the probe within the tumor target. Ablation was monitored with MR thermography. After completion of the ablation, we obtained an additional T1 MPRAGE sequence with half-dose gadolinium. The probe and RPD were then removed, and the Mini Bolt was capped under sterile conditions. The patient was returned to the operating table, while still on the AtamA® board. There, the Mini Bolt was removed, and the incision was closed with 3-0 Nylon suture. We then removed the patient’s head from the head-fixation ring.

Workflow 3 - Frameless stereotaxy, intraoperative MRI, no head fixation during ablation

This workflow is a salvage approach when head fixation during the laser ablation is not possible. Similar to workflow 2, the entire procedure for patient 6 was performed in the operating room with intraoperative MRI. The left posterior temporal location of the tumor target necessitated a lateral position. However, due to the patient’s girth, we were unable to position the head in the head-frame ring mounted on the AtamA® board. To overcome this problem intraoperatively, we used a Mayfield head clamp to obtain frameless stereotaxy with the BrainLab VarioGuide. No intraoperative CT was utilized. After placing the Mini Bolt and covering it with a sterile cap, the sterile drape was removed and the bolt was covered with sterile gauze. The Mayfield head clamp was released, and the patient’s head was rested on pillows. The patient was then transported to the intraoperative MRI. The head was taped to the table to prevent sudden movements. The remainder of this algorithm is similar to workflow 2, except that the patient’s head was not rigidly fixed during the ablation, but instead taped to the MRI table.

Intraoperative neurological assessment

The neurologic status of patients was intermittently assessed approximately every 5 minutes during the ablation, with emphasis on the modality most at risk due to its proximity to the tumor target and trajectory of the probe. Thus, speech was monitored in three patients, motor function in two, and vision in one. Motor monitoring was performed by asking patients to move the contralateral arm and leg to command. Speech monitoring included naming objects and asking patients to follow simple commands. Visual monitoring focused on testing visual fields via gross confrontation, with the physician reaching with his arm into the bore of the magnet.

Volumetric measurements

Estimates of targeted tumor volume were obtained with BrainLab software, while volumetric estimates of laser-ablated tissue were calculated on Monteris Neuroblate software. Volumetric estimates were concordant between the two software platforms.

## Results

**Figure 2:**
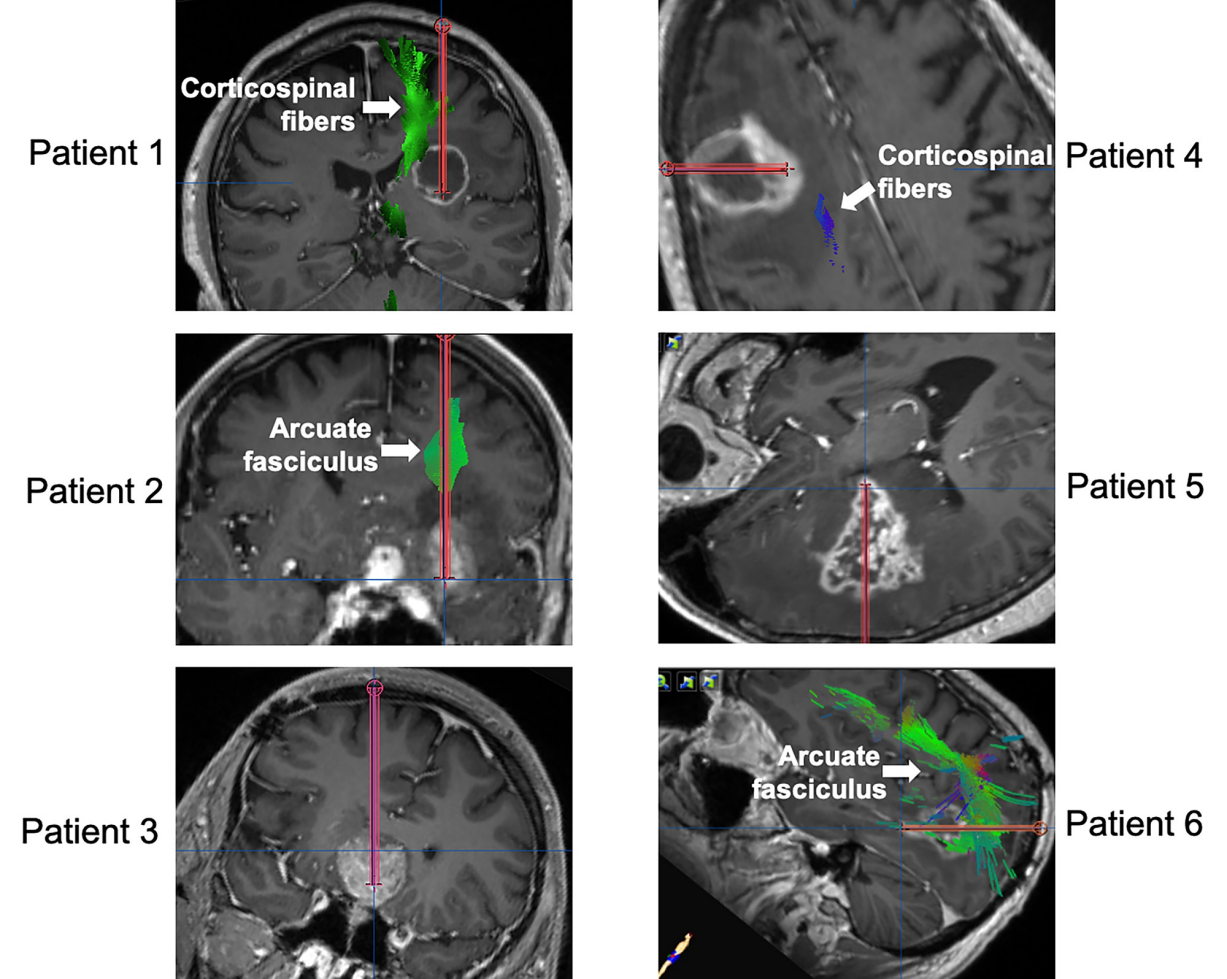
Preoperative imaging and planned trajectories. T1-weighted post-gadolinium images (“inline” or “trajectory” views) show the tumor targets and planned trajectories for each patient included in this study. Tractography identifies relevant fiber tracks in each case. For patient 3, the optic apparatus (chiasm and tracts) is not visible on this view, while for patient 5, the corticospinal tract is not visualized in this image (but can be appreciated in Figure 1).

Six patients were treated with awake LITT ablation of brain tumors using the NeuroBlate probe (Monteris) at our institution between July 2017 and May 2020 (Figure 2; Table 1). Five patients were male. The mean age was 60 years. The diagnoses included IDH (Isocitrate DeHydrogenase) wild-type glioblastoma (GBM) (Patients 1 and 6), IDH mutant oligodendroglioma (Patient 2), IDH mutant astrocytoma (Patient 5), melanoma metastasis (Patient 4), and pituitary adenoma (Patient 3). The neurologic modalities that we monitored included motor function (Patients 1,4,5), speech (Patients 2,6), and vision (Patient 3), because of proximity of tumor targets and probe trajectories to the corticospinal fiber tract, dominant arcuate fasciculus, and optic chiasm and tracts, respectively. The mean preoperative tumor volume targeted was 12.09 ± 3.20 cm^3^. The treated volume outlined by yellow TDT line (equivalent to 43 °C for 2 minutes) was 17.02 ± 4.07 cm^3^, while the volume outlined by the blue TDT line (equivalent to 43 °C for 10 minutes) was 12.06 ± 2.75 cm^3^.

**Table 1:** Demographic and clinical information

Patient Number	Age	Sex	Location	Diagnosis	Prior therapy	Pre-procedure neurologic baseline	Frame/head holder during probe placement	Stereotaxy	Head fixation during ablation	Laser Probe (Neuroblate, 3.3 mm)	Tumor Size (cm^3^)	Yellow TDT (cm^3^)	Blue TDT (cm^3^)	Neurologic function monitored	Relevant structure	Pre-procedure neurologic deficit	Post-procedure neurologic deficit	Length of stay (days)	Delayed deficit	Follow-up (months)	Clinical Outcomes
1	77	M	Left subinsular	IDH wild-type GBM	none	right hemiparesis	Leksell	frame-based (Leksell)	Leksell	diffuse tip	9.514	10.5	7.33	motor	corticospinal fiber tract	right hemiparesis (4/5)	no change	1	worsening right hemiparesis (3/5)	9	lost to follow up
2	74	M	Left inferior frontal	IDH mutant oligodendroglioma	none	gait instability, absence-like seizures, positional vertigo	Leksell	frame-based (Leksell)	Leksell	diffuse tip	4.333	7.91	5.98	speech	arcuate fasciculus	fluent speech	no change	1	no change	39	progressed at 15 months, alive at 39 months
3	42	M	Suprasellar / interhemispheric	Pituitary Adenoma (recurrent)	surgery, radiotherapy	right superior quadrantanopsia	Leksell	frame-based (Leksell)	Leksell	side-firing	6.689	9.39	6.13	vision	optic tracts	right superior quadrantanopsia	no change	1	no change	39	stable tumor size
4	76	F	Right frontal lobe	Melanoma	radiosurgery	left hemiparesis	Leksell	frame-based (Leksell)	Leksell	diffuse tip	12.514	17.6	13.8	motor	corticospinal fiber tract	left hemiparesis (4/5)	increased left lower extremity weakness (3/5)	4	worsening left hemiparesis (3/5)	3	expired after 3 months
5	34	M	Left frontal/basal ganglia	IDH mutant astrocytoma	surgery, radiotherapy, chemotherapy	intact	AtamA® System patient board - ring	Frameless (Brainlab Varioguide on head-fixation ring)	AtamA® System patient board ring	diffuse tip	26.587	23.1	16.6	motor	corticospinal fiber tract	intact	no change	1	none	6	pseudoprogression
6	57	M	Left posterior temporal	IDH wild-type GBM	surgery, radiotherapy, immunotherapy	word-finding difficulty, right homonymous hemianopsia	Mayfield	Frameless (Brainlab Varioguide on Mayfield clamp)	head taped to table	diffuse tip	12.887	33.6	22.5	speech	Wernicke's area/arcuate fasciculus	word-finding difficulty, right homonymous hemianopsia	no change	1	worsening speech	8	progression treated with additional radiotherapy

Only one patient (Patient 3) had his tumor ablated using a directional probe, in order to avoid injuring an azygos anterior cerebral artery encased by the tumor. The length of stay was one day for all patients, with the exception of one (Patient 4) who was discharged to rehabilitation on day 4. There were no complications related to the procedure in our cohort.

**Figure 3:**
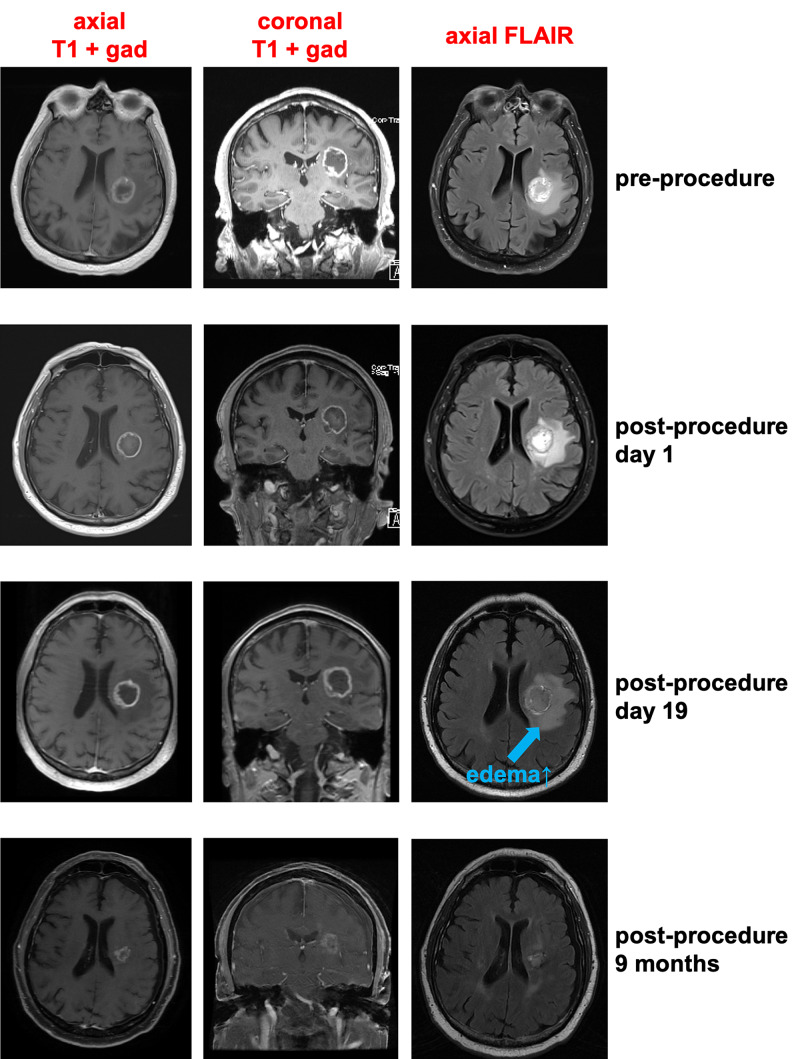
Delayed edema following laser ablation of tumors. We show relevant MRI images of patient 1, who underwent laser ablation of a left subinsular IDH wild-type GBM. Note that the peritumoral FLAIR signal is modestly increased in the MRI performed 19 days after the ablation. This was associated with worsening right hemiparesis that responded to dexamethasone therapy. Soon thereafter, the patient was treated with adjuvant chemoradiotherapy. Also note that nine months later, the enhancing tumor volume and peritumoral FLAIR signal have decreased significantly. That patient was being treated with bevacizumab at that time.

There were no intraoperative seizures or new neurologic deficits after the procedures. Patients were treated with moderate doses of dexamethasone postoperatively (typically 4 mg every 6 hours tapered over 7-10 days). One patient (Patient 4) experienced an acute postoperative mild deterioration in her baseline left hemiparesis that responded to increasing dexamethasone dosing. Three patients (Patients 1,4,6) suffered delayed deterioration of their pre-existing deficits 2-3 weeks after the LITT procedure and after completing their postoperative dexamethasone course (Figure 3). All delayed deficits responded to dexamethasone therapy.

## Discussion

We propose that, similar to conventional surgery, keeping carefully selected patients awake during LITT in eloquent brain areas ensures that heat does not injure perilesional cortical areas or subcortical fiber tracts, thus preventing neurologic deficits. While the intraoperative MR thermography and preoperative tractography information can help prevent heat spread to important fiber tracts, the frequent neurologic assessment during the ablation offers an additional layer of protection from neurologic injury.

Our patient cohort, while small and without a control group, shows that awake laser ablations are feasible and safe. Our approach toward awake LITT does not necessitate manufacturing a custom-made mask, which may delay scheduling the procedure and increase operative cost [15]. Instead, we utilize conventional head-fixation devices to immobilize the head along with local anesthetic, mild sedation and intravenous analgesics, as needed. Using this approach, our patients did not experience significant intraoperative discomfort.

Our study provides distinct workflow protocols that allow for awake LITT in either the diagnostic MRI suite or the intraoperative MRI environment. A technical issue to be discussed concerns workflow 3. We consider this paradigm a salvage solution when the patient’s head cannot be immobilized in a frame for the ablation. The technical limitation to be considered here is related to subtle head movements that occur during the ablation, while the patient is awake. Such movements result in a mismatch between the depiction of the TDT lines, as inferred from MR thermography, and the “registration” MRI image that the TDT contours are superimposed on. This problem can be mitigated by frequent re-registration of the patient using volumetric T1 images, which adds time to the procedure. Therefore, workflow 3 should be considered only in the event of unforeseen issues with head immobilization during the ablation.

An important observation in our cohort is delayed neurologic deterioration that occurs approximately 2-3 weeks after the ablation. We theorize that such delayed worsening of pre-existing neurologic deficits is likely due to increased cytotoxic edema and associated inflammation. Thankfully, this deterioration is transient and responds to corticosteroid therapy. Based on this experience, we monitor patient symptoms closely for three weeks post-procedure and adjust dexamethasone dosing accordingly.

## Conclusions

We propose that awake LITT in eloquent brain areas using conventional head-fixation devices is safe and may help prevent neurologic deficits.
